# On simulation, artificial intelligence, and the spirit of the Shokunin

**DOI:** 10.1186/s41077-026-00435-w

**Published:** 2026-04-10

**Authors:** Ben Symon

**Affiliations:** 1 Mater Misericordiae Limited, Mater Health Simulation Service, Mater Hospitals, Level 4, Duncombe Building, South Brisbane, QLD 4101 Australia; 2 Simulation Training Optimising Resuscitation for Kids (STORK) Statewide Simulation Service, Children’s Health Queensland, South Brisbane, QLD Australia; 3 Faculty of Medicine, University of Queensland, St Lucia, Brisbane, QLD Australia

**Keywords:** Artificial Intelligence, Simulation-based education, Cognitive debt, Professional identity, Craftsmanship, Shokunin, Reflection, Deliberate practice, Large Language Models, Healthcare education

## Abstract

**Background:**

The healthcare simulation community has rapidly embraced artificial intelligence (AI) and large language models (LLMs) for scenario design, delivery, and debriefing. While discussions have largely centred on efficiency and academic integrity, less attention has been paid to the philosophical and humanistic costs of this integration. This commentary explores the potential erosion of identity, expertise, and craftsmanship within simulation as cognitive labour is increasingly outsourced to machines.

**Main body:**

Drawing on the Japanese concept of the *Shokunin*—the devoted artisan in lifelong pursuit of mastery—the commentary contrasts the Western drive for efficiency with the intrinsic value of patient diligence and deliberate practice. Using the life and reflections of artist Katsushika Hokusai as a metaphor for the simulationist’s path, the author argues that expertise is cultivated through repetition, reflection and time—qualities at risk when AI automates creative and cognitive processes.

The concept of cognitive debt is introduced to describe the deferred mental cost of repeated reliance on AI, including diminished critical inquiry and weakened creative ownership. To preserve the spirit of the Shokunin, three guiding principles are proposed: (1) Know the grain of your wood before you carve—protect formative manual and reflective work early in one’s career; (2) Paint first with your own brush—prioritise original human creation before seeking AI feedback; and (3) Strive for personal growth—maintain curiosity and continuous growth as enduring antidotes to automation’s complacency. These principles urge simulationists to use AI as a supportive partner, not a surrogate for expertise.

**Conclusion:**

AI will undoubtedly enhance simulation’s technical capabilities, yet it also challenges our professional identity. To sustain meaning and mastery, simulation educators must intentionally preserve spaces for imperfection, repetition, and human creativity. The spirit of the Shokunin—humble, diligent, and ever-striving—offers a moral compass for navigating the evolving partnership between simulation and artificial intelligence.

**Supplementary Information:**

The online version contains supplementary material available at 10.1186/s41077-026-00435-w.

The healthcare simulation community is integrating Artificial Intelligence (A.I.) and Large Language Models into our design, delivery and debriefing processes at rapid pace [1]. Recent articles in Advances in Simulation and other prominent simulation journals have celebrated chain-prompted scenario design [2], real time scenario transcription and the potential for A.I. assistance with debriefing [3]. In our thirst for A.I’s potential to make our lives easier, discussion of negative impacts has focused primarily on the preservation of academic integrity [4]. Despite significant debate in other academic fields [5–8], the simulation community has reflected less about the cost of artificial intelligence on our identity, our self-worth, and our future as simulation craftsmen.

Exploring the spiritual costs of integrating A.I. into our work is important. As I watch my son’s 10 year old classmates secretly task ChatGPT with writing their poetry assignments, I note we face an unprecedented temptation to outsource thinking, creativity and learning. While A.I. represents a step towards a collective ‘transactive memory’, the path is bankrolled by sacrifices to personal growth [5] and human capacity [7]. Work can be tedious, but tedium teaches us. Outsourcing our work to A.I. comes with a ‘cognitive debt’ [8]: the chance to refine our own expertise and to reflect on our works with pleasure and pride.

Are we so eager to give this away, and if so, how much do we give? Without philosophical consideration on technology’s role within our workplaces, we risk becoming reductive cogs in an algorithmic healthcare system. While western culture emphasises efficiency and output, there exists an alternative frame in another culture: We could consider ourselves Shokunin.

## On Shokunin and Hokusai

The Japanese term ‘Shokunin’ (literally: artisan) has been used to describe a particularly devoted craftsman [9]. Regardless of the specific craft, be it wood carving, calligraphy or the creation of the perfect sushi meal, it can describe an individual entwined in relentless and lifelong pursuit of professional mastery. The term resonates with a call to humility, patient diligence, and the constant refinement of one’s skill in service to a greater communal purpose^1^.

Hokusai, a renowned Japanese visual artist from the Edo period, embodies many of the ideals of a Shokunin. Born in 1760, he created over 30,000 works in his lifetime including his masterpiece ‘The Great Wave Off Kanagawa’. It is a work of meticulous perfection, of vivid waves and muted skies, an incredible understanding of visual composition and design, and a masterful demonstration of a lifetime of deliberate practice. ‘The Great Wave’ has been celebrated, imitated and commercialised in a multitude of ways since its creation. It is alleged to have inspired Van Gogh’s ‘Starry Nights’ [10], Claude Debussy’s composition ‘La Mer’ [11], and triggered endless imitation, *japonisme* [12] and homage [13]. Hokusai’s cultural significance cannot be overstated.

**Fig. 1:**
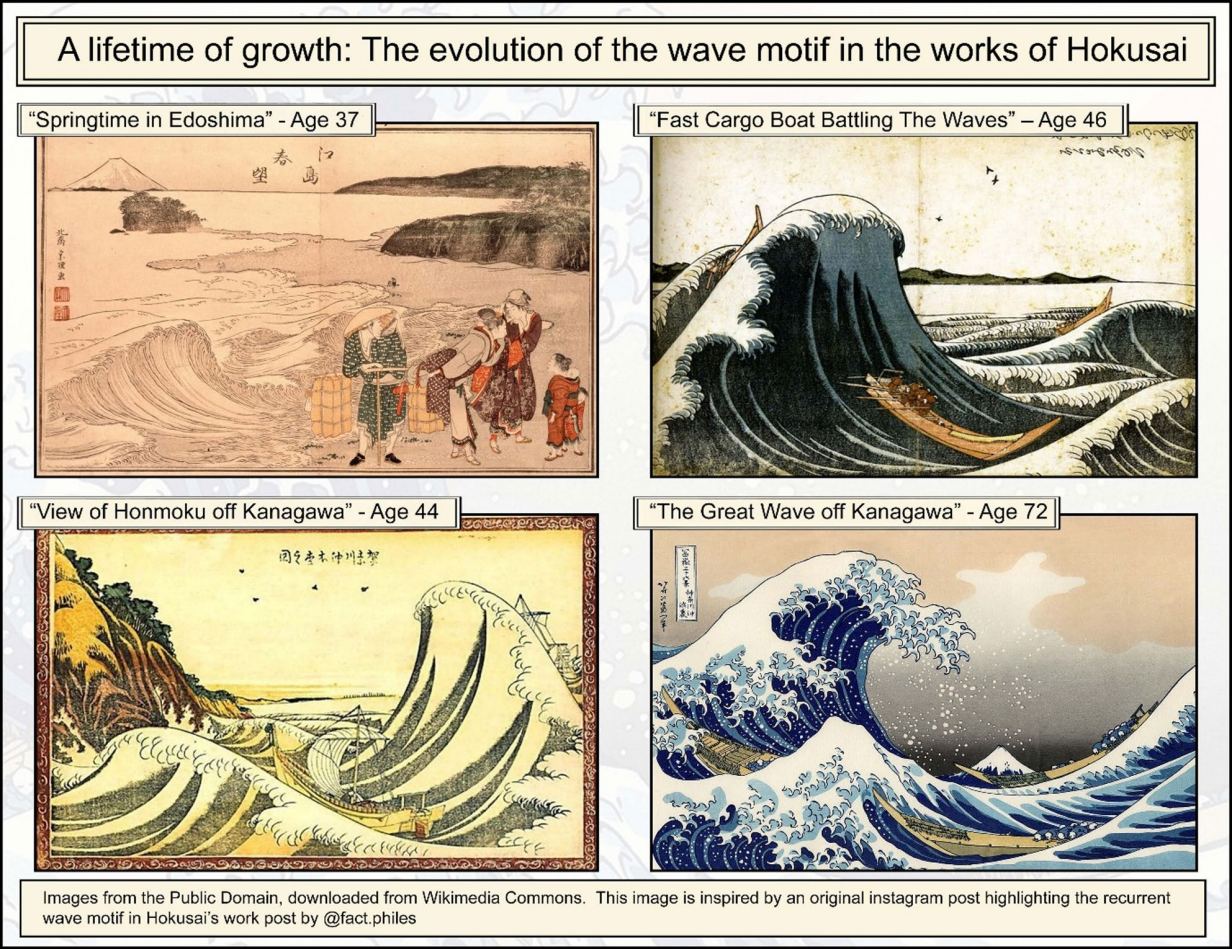
The evolution of the wave motif in the works of Hokusai, from estimated age 37 to 72

While Hokusai’s works are renowned, it is less well known that his most masterful pieces were created between the age of 70 until his death at 89, including the Great Wave. The wave itself was a recurrent motif in his work, gradually refined over a lifetime (see Fig. 1).

Hokusai’s older decades stand testament to the truth that expertise is cultivated through a lifetime of diligence. Within the notes of his book ‘One hundred views of Mt Fuji’, he echoes:

"...*until the age of 70*,* nothing I drew was worthy of notice. At 73 years I was somewhat able to fathom the growth of plants and trees*,* and the structure of birds*,* animals*,* insects and fish. Thus when I reach 80 years*,* I hope to have made increasing progress*,* and at 90 to see further into the underlying principles of things*,* so that at 100 years I will have achieved a divine state in my art*,* and at 110*,* every dot and every stroke will be as though alive. Those of you who live long enough*,* bear witness that these words of mine are not false.* [14]"

- Katsushika Hokusai, ‘One hundred views of Mt Fuji’, [translation by Henry D Smith II]

If we consider our careers in healthcare simulation from the lens of a Shokunin, I would argue our perspective on artificial intelligence may recalibrate. Like any profession, we can currently strive to master our craft; to continuously refine our approach to debriefing and reflection, and to labour towards a perfect simulated moment that meets the needs of our learners with effortless grace. As director of several simulation fellowships, I want future simulationists to feel pride, accomplishment, and the joy that comes from continuous improvement. I am not convinced that meaningful pride will come from an effortless series of prompts thrust into a large language model. While that loss is hard to measure, I believe it has meaning and is important for us to reflect on. As our species’ courtship with artificial intelligence progresses, we will need to negotiate clear boundaries between what we use that technology for, and how it impacts ourselves.

In their article ‘Your Brain on ChatGPT’, the authors describe the concept of cognitive debt: *“A condition in which repeated reliance on external systems like LLMs replaces the effortful cognitive processes required for independent thinking* [8]. *Cognitive debt defers mental effort in the short term but results in long-term costs*,* such as diminished critical inquiry*,* increased vulnerability to manipulation*,* decreased creativity. When participants reproduce suggestions without evaluating their accuracy or relevance*,* they not only forfeit ownership of the ideas but also risk internalizing shallow or biased perspectives.“.*

While this concept does not preclude using A.I., it should be folded into our evaluations of its impact on both our work and ourselves. If the cost of outsourcing your work to A.I. is your own expertise, how will you ‘reinvest’ in your own performance to equilibrate the cognitive debt?

How then, to avoid losing the spirit of the Shokunin? I share 3 principles to implement in our practice, to retain expertise, pride and ownership of our work.

## Know the grain of your wood before you carve

“The path to greatness requires hard work, discipline, and repetition” [9].

A Shokunin’s path to expertise is built through repetition and work. Describing apprentice chefs for a sushi restaurant, Holt and Yamauchi describe “*during the early years they barely get to touch the food*,* spending more time with a sweeping brush and detergents than with chopping boards and ingredients. As apprentices they learn by endlessly preparing the rice*,* each iteration constituting nothing more than the difference of its being the next in a long sequence through which conscious rule following becomes thoughtless expertise*,* then it’s the eggs for the omelettes*,* then the rolling of seaweed. Again and again*.” [15].

From a western lens, this ritualistic process could be seen as excessive, inefficient or gratuitous, however healthcare simulation has long recognised the importance of repetition in learning. Rapid Cycle Deliberate Practice harnesses intensive periods of deliberate practice for longer skill retention [16], while the Helping Babies Breathe program has explored the impact and timings for optimal spaced practice [17]. Regardless of the efficiencies offered by A.I., it is essential that we recognise that repetitive work helps us to refine our practice through myriad micro-variations and the subsequent reinforcement of neural pathways. Particularly early in our career, crafting scenarios and debriefing scripts by hand is likely to be more beneficial to our development than feeding a few prompts into A.I and adjusting some sentences. A simulationist in training will likely benefit from the work found in deliberate multi-disciplinary cross-consultation, careful consideration of learning goals, and the conscious titration of observations and patient descriptions. I would encourage simulation leaders to ensure our trainees continue to immerse themselves in this work, and A.I. tempts us to sidestep many of these processes.

## Paint first with your own brush

If we prompt A.I. to create a simulation, it will likely reflect a reductive conglomeration of what has already existed. This limits creativity, innovation and our own sense of pride. I would suggest taking a more effortful path, embracing the cognitive work of crafting something by hand. Write your sims by hand, engage in co-creation with other professions, and embrace the fact that a slower process builds one’s own expertise.

Once you have refined your work, *then* share it with A.I. software with a deliberately critical lens. Prompt it not to rewrite, but instead to give you feedback and advice. This allows you to maintain control emotional and intellectual ownership if your work, and to grow your expertise. In this digital supplement, I share a conversation with ChatGPT where I attempt to gain coaching on this essay, defining boundaries in our conversation to ensure I retain ownership and voice, requesting critique rather than rewrites, and asking it to take opposing stances on the commentary itself.

If workplace efficiencies demand A.I. integration, I suggest liberalising one’s use of A.I. most freely on tasks you have already mastered, or that are less likely to contribute to one’s professional development.

## Strive for growth

There has been no greater professional joy in my life than gradually learning about simulation, debriefing and quality improvement science [18]. In our monthly podcasts on Simulcast, for almost 10 years I have had the chance to share knowledge, reflect, and learn with my colleague Victoria Brazil and the broader healthcare simulation community. That journey has given me the incredible privilege of regular refinement and reflection. A little bit of new knowledge, every month, slowly accumulating over time. It is intoxicating. Few things have protected me from professional burnout more than the opportunity for incremental growth and reflections with a dear and trusted friend.

In the documentary ‘Jiro Dreams of Sushi’ [19], 85 year old sushi master Jiro Ono states “*All I want to do is make better sushi. I do the same thing over and over*,* improving bit by bit.*

*There is always a yearning to achieve more. I’ll continue to climb*,* trying to reach the top… but no one knows where the top is. Even at my age*,* after decades of work… I don’t think I have achieved perfection. But I feel ecstatic all day… I love making sushi. That’s the spirit of the shokunin.”.*

Whatever technological choices we make in the future, we must retain the shokunin’s spirit to strive for personal growth and towards perfection.

## Conclusion

A.I. will inevitably integrate into our lives in the future, and it may yet make the world a better place. All the same, I urge our community to carve protected spaces in academia for imperfect works that are exclusively generated by human endeavour; To reflect on the value of repetition, time and incremental adjustments in our professional journeys; To define boundaries in our relationship with A.I. that ensure we are strengthened and empowered by it, rather than cognitively weakened. And most importantly, to remember the beauty of a life full of human error, of tedium, and the joy of eternally learning.

## Supplementary Information

Supplementary Material 1.

## Data Availability

No data utilised in this commentary. Images utilised are in the public domain and their files sourced from Wikimedia Commons.

## Abbreviations

AI: Artificial intelligence
LLM: Large language model

## Footnotes

1 The author would like to acknowledge that the term ‘Shokunin’ has multiple interpretations and at times the term has been accused of being used by Westerners with an air of Eastern romanticism. The term is also used in common parlance to describe a craftworker without any additional cultural implications.
